# Genome Sequence of *Lawsonia intracellularis* Strain N343, Isolated from a Sow with Hemorrhagic Proliferative Enteropathy

**DOI:** 10.1128/genomeA.00027-13

**Published:** 2013-02-28

**Authors:** Michelle Sait, Kevin Aitchison, Nick Wheelhouse, Kim Wilson, F. Alex Lainson, David Longbottom, David G. E. Smith

**Affiliations:** a Institute of Infection, Immunity and Inflammation, College of Medical, Veterinary and Life Sciences, University of Glasgow, Glasgow, United Kingdom; b Moredun Research Institute, Pentlands Science Park, Bush Loan, Edinburgh, Midlothian, United Kingdom

## Abstract

*Lawsonia intracellularis* is the etiological agent of proliferative enteropathy (PE), causing mild or acute hemorrhagic diarrhea in infected animals. Here we report the genome sequence of strain N343, isolated from a sow that died of hemorrhagic PE. N343 contains 24 single nucleotide polymorphisms and 90 indels compared to the reference strain PHE/MN1-00.

## GENOME ANNOUNCEMENT

*Lawsonia intracellularis* is a Gram-negative, obligate intracellular bacterium which has been identified as the causative agent of proliferative enteropathy (PE). PE is endemic in pigs, causing gross pathological changes to the lower intestine through hyperplasic lesions and a thickening of the mucosal tissue (1). Symptoms include chronic, mild diarrhea or acute hemorrhagic diarrhea resulting in poor growth rates, weight loss, or in extreme cases, death (1). Recently, it was shown that host susceptibility to *L. intracellularis* infection depended on the species of origin (2), highlighting the need for additional genome sequences to facilitate our understanding of the mechanisms of infection and pathogenesis. Here we report the genome sequence of *L. intracellularis* N343, which was isolated from the ileum of a sow from a farm in Minnesota that died of hemorrhagic PE.

*L. intracellularis* N343 was propagated in Int407 cells at 37°C under microaerophilic conditions (8.8% CO_2_, 8.0% O_2_) (3). Bacterial cells were purified by density gradient centrifugation, and genomic DNA was extracted using a DNeasy blood and tissue kit (Qiagen). Genome sequencing and assembly were performed commercially by Beckman Coulter Genomics (Danvers, MA). Briefly, a standard genomic library was sequenced using an Illumina HiSeq 2000 sequencer to generate 202,176,161 100-bp single-end reads. Following filtering of reads against pig and human reference sequences, a reference-guided assembly of 1,679,789 reads was performed against *L. intracellularis* PHE/MN1-00 (NC_008011, NC_008012, NC_008013, NC_008014) using Mira (4). Assembled reads provided an average 13× sequence coverage across the entire genome. Gaps were closed by PCR and sequencing. Annotation was performed using Artemis (5).

*L. intracellularis* N343 is composed of a 1,457,568-bp circular chromosome with a G+C content of 33.2%, with 1,182 predicted coding sequences (CDSs). There are 3 plasmids that are 27,133 bp, 39,878 bp, and 194,613 bp in size, with G+C contents of 29.0, 29.2, and 32.9% and containing 29, 24, and 104 predicted CDSs, respectively. There are two rRNA gene operons and 44 tRNA genes representing all standard amino acids except pyrrolysyl. Comparative analysis between N343 and PHE/MN1-00 identified 8 single-nucleotide polymorphisms (SNPs) and 70 insertion/deletions (indels) in intragenic regions, and 16 SNPs (3 synonymous, 13 nonsynonymous) and 20 indels in intergenic regions.

The genomic features of *L. intracellularis* N343 include the genes required for glycolysis, the pentose phosphate pathway, and the biosynthesis of peptidoglycans and lipopolysaccharides. However, components of the tricarboxylic acid (TCA) cycle and those required for the synthesis of specific amino acids are absent. Strain N343 contains 57 genes involved in ATP binding cassette (ABC)-type transport systems. Two-component signal transduction systems involved in sensing nitrogen availability and chemotaxis are present, as are the genes required for flagellum assembly. A wide range of genes related to virulence, adhesion, and invasion were identified, including genes predicted to be involved in type III secretion and genes belonging to the type V autotransporter (AT) protein family. Of these, two classical ATs were located on the chromosome, with the remaining located on plasmids 2 and 3.

### Nucleotide sequence accession numbers.

Genome and plasmid sequences are available at DDBJ/EMBL/GenBank under accession numbers CP004029 (chromosome) and CP004030, CP004031 and CP004032 (plasmids 1 to 3).
